# PILOT RCT TO EXAMINE A MOBILE HEALTHY EATING INTERVENTION FOR PERSONS WITH FRAILTY

**DOI:** 10.1093/geroni/igad104.1843

**Published:** 2023-12-21

**Authors:** Oleg Zaslavsky, Kuan-Ching Wu, Aishwarya Naik, Yan Su

**Affiliations:** University of Washington, Seattle, Washington, United States; University of Washington, Seattle, Washington, United States; University of Washington, Seattle, Washington, United States; Illinois State University, Normal, Illinois, United States

## Abstract

New scalable approaches are needed to promote healthy eating and improve health in persons with frailty. We evaluated a new mobile intervention consisting of a patient-facing app and clinician interface to support Mediterranean-style eating in a pilot RCT. Fourteen females and one male, averaging 71 years of age, were randomized to intervention (n=8) or control (n=7) for three months. In the intervention, participants were encouraged to interact with the app at least once a week, whereas, in control, participants received handouts with information and online resources concerning the Mediterranean Diet. Mann-Whitney and Cohen’s d estimates were used to compare groups and evaluate effects. All participants completed and consistently adhered to the study protocol throughout the study. At the end of the study, there was a significant improvement in the intervention compared to the control in the following outcomes: SPPB (W=45, p=0.049; d=1.14); Mediterranean Diet Knowledge (W=49.5,p=0.013; d=1.26); consumption of vegetables (W=47, p=0.03; d =1.42); consumption of legumes (W=54, P=0.001; d=1.32), and negative outcome expectations (W=9,p=0.03; d=0.56). Albeit insignificant, intervention group participants improved their inflammatory, lipids, and glycemic blood measures. At the exit interviews, most participants expressed satisfaction with the app and acknowledged that the app was useful and easy to operate. They also provided points for improvement concerning control over the frequency of interactions with the app, more personalized choices for meals, and better visualization of their progress. The study demonstrated the preliminary efficacy of the digital intervention and suggested several mechanistic measures involved in behavioral change.

